# Self-care Ability of Older People Living in Urban Areas of Northwestern Iran

**Published:** 2018-12

**Authors:** Jafar Sadegh TABRIZI, Mehrdad Amir BEHGHADAMI, Mohammad SAADATI, Ulrika SÖDERHAMN

**Affiliations:** 1. Tabriz Health Service Management Research Centre, Tabriz University of Medical Sciences, Tabriz, Iran; 2. Dept. of Health Services Management, School of Health Service Management and Medical Informatics, Tabriz University of Medical Sciences, Tabriz, Iran; 3. Iranian Center of Excellence in Health Management, School of Management and Medical Informatics, Tabriz University of Medical Sciences, Tabriz, Iran; 4. Student Research Committee (SRC), Tabriz University of Medical Sciences, Tabriz, Iran; 5. Road Traffic Injury Research Center, Tabriz University of Medical Sciences, Tabriz, Iran; 6. Center for Caring Research, Southern Norway, Faculty of Health and Sport Sciences, University of Agder, Grimstad, Norway

**Keywords:** Self-care ability, Elderly, Health complexes, Iran

## Abstract

**Background:**

Self-care ability was considered as a mechanism to improve the quality of life and reducing the high costs of medical services for elder people. Iran is experiencing increased elder people and healthy aging is one of the challenges. This study aimed to investigate self-care ability of elder people in Shahid Chamran and Dr. Shadpour health complexes, Tabriz- Iran.

**Methods:**

This was a cross-sectional study done in 2015. “The Self-care Ability Scale for the Elderly (SASE)” valid and reliable questionnaire (Persian version) was used for data collection. Totally, 220 samples were selected using G-Power 3.1.5 software. Sampling was conducted through systematic random method. Data were analyzed using SPSS 21 software. Descriptive statistics and independent t-test, one-way ANOVA, Mann-Whitney, Kruskal-Wallis test and Kolmogorov-Smirnov test were used.

**Results:**

The mean of self-care ability in elder people was calculated as 56.69± 15.07 (out of 100). 96.8% of participating elderlies, had poor self-care ability. Significant relations were found between elder people self-care ability and their educational level, life status, employment status, and marital status (*P*<0.05). However, gender, income source, and insurance status had no relations with self-care ability of the elder people (*P*>0.05).

**Conclusion:**

Self-care ability of elder people in Tabriz was at weak level. Regarding, health providers must employ the programs to promote older people self-care ability including improving self-esteem, receiving family help, improved nutrition and mental health improving elderly’s quality of life.

## Introduction

Aging is one of the phenomenon in the global health area (1) and it was considered as one of the natural stages of human life from birth to aging took place over the time (2). Dynamics of biological, perception, development and maturation processes (3) in aged people led to physiological and social changes (4) in which people lose their independence due to physical and mental disabilities and many other causes (5). Increasing population of elder people is a phenomenon of both developed and developing countries (6) and many nations had considered it as the phenomenon of the 21st century (7). The population of elder people has been estimated at over 605 million. This figure to reach two milliard people in 2050 higher than the children population growth (8). Iran is not an exception to this. The number of older people (upper than 60 yr old) in Iran was about 4560000 people including 6% of Iran total population in 2006 (9) reached to 8.2% in 2014 (10). Healthy aging is the right of all human beings, and this increases the importance of the aging phenomenon and prevention of its problems in Iran. Considering, it is required immediately planning to provide services for this class of people in society to prevent this problem (11).

The ability to take care of elder people is very important source of health and it might be considered as a determining factor to manage everyday lives of elder people. Due to aging, self-care ability reduces in many people because of one or several factors, leading to a decrease in the life satisfaction of the elder people. As a result, gaining insight and understanding of the influential factors will be important (12). Self-care has been defined as a multidimensional concept related to health that is interpreted in different ways in the literature (13). Considering definitions and correct understanding of the self-care concept, depending on professional theoretical and philosophical approach, there is no consensus in this regard. However, one tool, especially to evaluate self-care ability in elder people, the Self-care Ability Scale for the Elderly (SASE) (14, 15). Self-care means a set of information, willingness to self-care and self-care skill in elder people (16). Individuals have the ability and the power to take all necessary care for health (17). Regarding, human had been considered as own self-care responsible for performing all cares required to health in order to maintain his life, welfare, and well-being, to feel happiness permanently (18). Self-care agency is defined as the ability to perform self-care activities that individuals perform them to maintain, restore or improve their own health (19). The construct of self-care ability is grounded in the belief that human beings are rational acting subjects, have their own free will and can act in relation to themselves and other individuals(15). A person’s self-care ability is the capacity to care for oneself and may be considered as the power or the potential for self-care actions, that is a necessary condition for these actions (15, 20).

In a Swedish study, self-care ability decreases with aging (21). Self-care ability was reduced in older people. In addition to age, disability and the need to receive help was considered as a risk factor for low self-care ability among the elder people (21, 22). In a meta-analysis study on self-care behavior by elder people in Thailand, Klainin and Ouannapiruk (2010) showed that demographic variables such as age, gender, and education had not a desirable relationship with self-care behavior (23). On the other hand, self-care ability of elder people was associated with receiving health service (24). Increased self-care ability in elder people could lead to self-actualization. Many healthcare organizations and providers had considered self-care ability as a mechanism to reduce the high costs of medical services and thus to improve the health of individuals and society (25). Considering, the growing trend of aging in Iran, it is a necessity to investigate self-health and the way in which older people take care of own health.

This study was conducted to investigate self-care ability of urban living elder people in Shahid Chamran and Dr. Shadpour health complexes, Tabriz -Iran.

## Methods

This cross-sectional study was conducted in Shahid Chamran and Dr. Shadpour health complexes in Tabriz, Iran 2015. Health complexes are new health facilities in which PHC services are provided with some professional services. Each health complex contains 4–5 health centers covering 10000–30000 people. Health centers, generally, provide PHC services and in a need, refers the people for professional services to health complex clinics. Shahid Chamran health complex is in suburban area of Tabriz located at seventh region of municipality and at 3 km southwest of this city. It has lower socioeconomic status than other parts of Tabriz. In return, Shadpour Health complex is located in Zafaranieh district of Tabriz that is one of the areas with high socioeconomic situation in Tabriz.

### Sample

Study population consisted of all elder people who had health file in Shahid Chamran and Dr. Shadpour health complexes of Tabriz. Totally, 220 samples were calculated to include in the study using G-Power 3.1.5 software. Sample allocation to each center was equally. Sampling was conducted by systematic random method using list of elder people.

### Data collection

The questionnaire had consisted of two parts, first, demographic characteristics of elder people and the second17-item self-care ability of elder people questionnaire (SASE) (15, 26). Response to each question was categorized based on a 5-point Likert scale. The highest score was 100 and the lowest score was 20. Dimensions of self-care ability include the ability to take care of personal responsibility, the ability to take care of goals, and ability to take care of health (15, 20). Questionnaire was translated to Persian language using Backward-Forward method by experts in English language and Elderly health. The validity and reliability of the questionnaire were confirmed using experts panel and a pilot study (n=30), respectively (CVI=0.96, α=0.73). Data were gathered through self-completion of the questionnaires by elderlies. Every elderly who was illiterate or was not able to read, the questions were red by the researchers and the answers were ticked.

### Data analyzing

Data were analyzed using SPSS 21 software (Chicago, IL, USA). Descriptive statistics (Mean and SD) were used to report self-care ability status in elder people. Moreover, independent t-test was used to compare self-care ability among elder people, and two-state (binary) independent variables (such as male and female) and ANOVA were used to compare self-care ability according to more than two states variables (such as academic groups). Kolmogorov-Smirnov test was used to investigate the normality of data and non-normal data were analyzed using the Mann-Whitney and Kruskal-Wallis test. With an adoption on Söderhamn et al studies (14, 15), the self-care ability mean higher than 80, was classified as good self-care ability, and the mean lower than 80 were classified as poor self-care ability.

### Ethics

The study was approved by the Ethical Committee of Tabriz University of Medical Science.

## Results

The mean age of samples was 68.65± 7.17 yr and 52.3% of them were female. In addition, 58.6% of the elder people were married, 51.8% of them were illiterate, 39.1% of them had social security insurance, and 48.2% of them had complementary insurance. Additionally, 56.4% of them were living with their spouses and income source of 41.8% of them was pension. The mean of self-care ability in urban living elder people of Tabriz was 56.69± 15.07 (Table 1). 96.8% of participating elderlies, had poor self-care ability (14, 15). According to independent *t*-test results, significant relation was found in self-care ability of elderly’s belonging to each of health complexes. The mean of self-care ability and its dimensions in elder people of Chamran Health Complex were higher compared to Shadpour Health Complex elderlies (Table 2). Significant difference was found between elder people self-care ability and their educational level, life status, employment and marital status (*P*<0.05) (Table 3).

**Table 1:** Mean, SD for self-care ability and dimensions

**Statistical indicators**	***Self-care ability and its dimensions***			
	***Self-care ability***	***Ability to take care of personal responsibility***	***Ability to take care of goals***	***Ability to take care of health***
Mean	56.69	59.60	53.86	54.60
SD	15.07	18.03	18.68	27.85

**Table 2:** Comparison of self-care ability and dimensions of elderly people in Shahid Chamran and Dr. Shadpour Health complexes

***Self-care ability and dimensions***	***Complex***	***Mean***	***SD***	Sig^*^
Self-care ability	Chamran	59.91	14.34	0.355
	Shadpour	53.46	15.35	
Ability to take care of personal responsibility	Chamran	62.18	15.21	0.033
	Shadpour	57.01	20.21	
Ability to take care of goals	Chamran	57.04	20.18	0.011
	Shadpour	50.68	16.55	
Ability to take care of health	Chamran	60.56	19.68	0.007
	Shadpour	48.63	33.15	

* *P*-value is based on t-test.

**Table 3:** Self-care ability relation with demographic characteristics of the elderly

***Statistical Indicators***		***Self-care ability***		

		***Mean***	***SD***	P-Value*
Educational level	Illiterate	47.68	16.29	0.004
	Elementary	54.04	15.71	
	Diploma	61.38	17.17	
	Graduate	52.02	18.64	
	Master degree	47.47	18.41	
Marital status	Married	64.20	22.22	0.003
	Single	59.41	13.42	
	Divorced	61.02	21.68	
	Death of spouse	53.87	18.01	
Employment status	Housewife	60.88	20.90	0.049
	Unemployed	58.36	14.27	
	Retired	55.47	15.39	
Life status	With spouse	57.72	14.04	0.005
	Alone	60.81	15.01	
	With children	52.48	17.68	

* *P*-value is based on one-way ANOVA.

According to LSD Post-Hoc multiple comparisons results, significant differences were appeared between elder people educational level and their self-care ability, as it was different in elder people who were illiterate and those who had associate, graduate, and master degree. In marital status variable, significant correlation was found between elder people married and those who were living with spouse and their self-care ability. In addition, in the variables of employment, significant relationship was found between elder people retired and unemployed and their self-care ability. In the variable of life status, significant correlation was observed between elder people with spouse and children variable (vice versa) and between alone and with children elder people variable in self-care ability of elder people.

## Discussion

The aging issue of world population is a relatively new phenomenon. Therefore, to cope with the challenges of this phenomenon, adopting appropriate policies in order to improve the elder people status is very important and it has been included in the agenda of international organizations (27). Self-care ability of urban living elder people in Tabriz was estimated at weak level (56.69± 15.07). Similarly, self-care ability dimensions were estimated at the weak level in the elder people.

Significant difference was found between elderlies of health complexes in the self-care ability and its dimensions so that self-care ability in elder people of Shahid Chamran Health Complex was higher than that in elder people of Shadpour Health Complex. As the socio-economic status of the elder people was lower in the Shahid Chamran Health Complex, this can affect their self-care ability. People who have lower financial power showed greater willingness to use preventive measures (including traditional medicine) and elder people are not exception in this regard (28). This might be the cause of their more power to maintain their health and higher self-care ability. This result outstands the necessity of social status consideration in public health policies development (28).

Factors such as age, gender, type of insurance, complementary insurance, and source of income had no significant correlation with self-care ability of elder people (*P*>0.05), but other factors such as education, life status, employment and marital status had significant relation with self-care ability of elder people (*P*<0.05). Males had higher self-care ability than females, while significant difference was not reported in self-care ability of two genders statistically (29). Male elder people self-care ability was higher than that of females, but this difference was not significant (30). Results of these two studies are consistent with findings of our study. The effect of gender difference on self-care ability can be affected by other variables such as level of knowledge and physical, psychological and behavioral status of people. Better self-care ability of male people is due to higher educational level of male people participated in the study compared to female people (31). Self-care ability and activity are learned behaviors that an individual should learn them since his childhood to adulthood, and formal education, as basic influential factor, has direct relationship with self-care ability (32). Additionally, higher education affects self-care ability of people due to its relationship with better job opportunities and higher income (29). Elder people with higher educational level have better judgment power and decision making to perform self-care behaviors (33). Our study results were consistent with these literature results.

A significant relation was showed between marital status and self-care ability of elder people so that self-care ability of married people was higher than that of other people (30). This result is in line with result of our study. Being married could affect self-care ability of elder people due to the role that spouse could play in reducing the old age period through emotional supports and help to lifestyle (29). However, loneliness of elder people did not emerge as an associated factor with elderlies self-care ability (12). Regarding the current study results, alone elderlies had the most mean of self-care ability rather than others. This may due to the low number of alone elderlies participating in the study.

The study results present a valuable information about elder people self-care ability regarding geographically and socio-economically different contexts.

The questionnaires were filled via self-reported information by elderlies and may be over or under-response. Moreover, any generalization must be cautious, due to the study design and population.

## Conclusion

Generally, self-care ability of urban living older people in Tabriz was at the weak level. Therefore, preventing risk factors of self-care ability that is inability perception, receiving help from family, the risk of malnutrition and disorder in mental health, and strengthening self-care ability factors such as being active, preparing your own food, the ability to find goal in life are very important in this regard.

Elder people health policy-makers should have knowledge on the way to express this ability and they should be aware of their responsibility to identify and design need for appropriate support and help and preventing unnecessary and unwanted dependencies of the elder people. They should also adopt appropriate measures to improve self-care ability of elder people.

## Ethical considerations

Ethical issues (Including plagiarism, informed consent, misconduct, data fabrication and/or falsification, double publication and/or submission, redundancy, etc.) have been completely observed by the authors.
